# Acute fibrinous and organizing pneumonia after bone marrow transplantation, an underrecognized, severe condition

**DOI:** 10.1016/j.htct.2025.106238

**Published:** 2026-01-10

**Authors:** Anita Cassoli Cortez, Maria Cristina Nunez Seiwald, Ana Rita Brito Medeiros da Fonseca, Aliana Meneses Ferreira, Gabriella Pogorzelski, Leonardo de Abreu Testagrossa, Yana Novis, André Nathan Costa

**Affiliations:** a Bone Marrow Transplantation Department, Sírio Libanês Hospital, São Paulo, Brazil; b Pulmonology Department, Sírio Libanês Hospital, São Paulo, SP, Brazil; c Division of Pulmonology, Instituto do Coração (Incor), Hospital Das Clínicas HC-FMUSP, Faculdade De Medicina, Universidade De São Paulo, Brazil; d Department of Pathology, Hospital Sírio-Libanês, São Paulo, Brazil

**Keywords:** Graft-versus-host disease, Bone marrow transplantation, Acute fibrinous organizing pneumonia

## Abstract

**Introduction:**

Allogeneic bone marrow transplantation can lead to various pulmonary complications, including acute fibrinous organizing pneumonia. This condition is rare and presents with aggressive clinical features, distinct from other forms of organizing pneumonia, such as cryptogenic organizing pneumonia.

**Method:**

A literature review using the PubMed, Embase, Lilacs, and Cochrane databases was conducted to analyze cases of acute fibrinous organizing pneumonia following transplantation focusing on clinical features, therapeutic approaches, and outcomes.

**Case Report:**

The case of a 62-year-old female who developed acute fibrinous organizing pneumonia after transplantation for acute myeloid leukemia is presented. Despite an initial absence of infectious agents, parainfluenza virus was later identified in a bronchoalveolar lavage. The patient progressed to severe hypoxemic respiratory failure and was unresponsive to corticosteroids and rituximab, ultimately dying seven months post-transplant.

**Conclusion:**

This is a rare and severe complication following allogeneic bone marrow transplantation. Early diagnosis, histopathological confirmation, and prompt initiation of corticosteroid therapy are critical for improving outcomes. Patients diagnosed before Day +100 generally have a better response to treatment and more favorable clinical outcomes. The need for a more effective and targeted treatment strategy remains an unmet challenge in managing this condition.

## Introduction

Long-term disease-free survival and the possibility of cure are goals for patients who undergo allogeneic bone marrow transplantation (allo-BMT). However, despite improvements in supportive care, early and late transplant-related complications remain a significant cause of morbidity and mortality.

Pulmonary infectious and noninfectious complications are reported in 30–60 % of all allo-BMT recipients and carry high morbidity and mortality rates [1,2].

Pulmonary complications encompass a heterogeneous group of conditions including graft versus host disease (GvHD), frequently manifested as bronchiolitis obliterans (BO), organizing pneumonia (OP) – which in the post-bone marrow transplant context is also known as cryptogenic organizing pneumonia – pulmonary edema, diffuse alveolar hemorrhage and idiopathic pneumonia syndrome [1].

Acute organizing fibrinous pneumonia (AFOP) is a rare presentation of acute lung injury that resembles OP. This disease was first described in 2002 as a unique histological pattern of acute lung injury that is histologically different from diffuse alveolar damage, eosinophilic pneumonia [3]. Although the pathology of diffuse alveolar damage and OP may be similar to AFOP, these are distinct conditions [4].

In lung transplant patients, chronic lung allograft dysfunction shares striking similarities with the pathogenesis and underlying immunopathology of lung GvHD after allo-BMT [5]. AFOP has been identified as a novel form associated with a significantly poorer prognosis than other forms of allograft dysfunction [4,6].

In this report, we present a case of AFOP following allo-BMT, potentially exacerbated by parainfluenza virus infection, which was refractory to both corticosteroids and rituximab. Furthermore, our aim was to review the characteristics of the major non-infectious organizing pneumonias that occur post-bone marrow transplantation (Table 1). Additionally, a literature review of AFOP following allo-BMT is provided, detailing the therapeutic approaches employed and the outcomes of each case (Table 2).

**Table 1:** Characteristics of non-infectious pneumopathies after bone marrow transplantation.

**Non-infectious pneumopathy**	**Pathological findings**	**Radiological findings**	**Clinical course**
Organizing pneumonia	Loose organizing fibroblastic plugs: loose plugs of fibromyxoid tissue within alveolar ducts, surrounding alveoli, and focal peribronchiolar and mild-to-moderate interstitial chronic inflammation	Single or multiple, sometimes migratory alveolar opacities; ground-glass pattern; consolidations; reversed halo sign	Pneumonia-like presentation with fever, malaise, cough, and dyspnea. Usually responsive to corticosteroids, in some cases immunosuppressants may be needed. Can be recurrent
Acute fibrinous organizing pneumonia	Reactive pneumocytes and presence of fibroblastic plugs associated with intra-alveolar fibrin deposition, often organized into balls. Type II pneumocyte hyperplasia. Prominent fibrin deposition in alveolar spaces and sparse granulation tissue in the form of fibromyxoid plugs	Diffuse bilateral opacities; consolidations; nodular opacities; more diffuse distribution than classic organizing pneumonia	Acute or subacute: fever, cough, progressive dyspnea, hypoxemia, rapid progression in some cases. Poorer outcome, high mortality, may have fulminant course, unresponsive to high dose corticosteroids and immunosuppression

**Table 2:** Reports of acute fibrinous organizing pneumonia (AFOP) after allogeneic bone marrow transplantation between 2009 and 2024.

**Author**	**Age Gender**	**Diagnosis**	DAT AFOP presentation	**Conditioning/ GvHD prophylaxis**	**GvHD**	**Concomitant lung infection**		**Ventilatory support**	**AFOP treatment**	**Outcome**
Lee SM et al. 2009	60 /M	Acute myeloid leukemia	4 months	Fludarabine and Melphalan/cyclosporine (cyclosporine (Cyclosporine capsules) capsules) and methotrexate (Methotrexate) (Methotrexate)	NA	No		OI	Methylprednisolone 45mg/d for 30 days	Death
Nguyen et al. 2016	39 / M	B-cell lymphoblastic leukemia	6 weeks	NA	NA	No		OI	Prednisone 1mg/kg	Resolution after 6 months
Nguyen et al. 2016	25 / M	B-cell lymphoma	25 days	NA	NA	No		OI	Prednisone 1mg/kg	Resolution after 6 months
Simmons, GL et al. 2017	52 / M	Angioimmunoblastic T cell lymphoma	*D* + 325	ATG+TBI 4.5Gy/Tacrolimus and MMF	Mild chronic GvHD skin and eyes	No	OI		Methylprednisolone 250 mg q6h+ Tacrolimus + Etanercept 25 mg twice weekly – 8 doses	Resolution after 40 days Remain in CR 2.5 years after BMT
Present case	62/ F	Acute myeloid leukemia	*D* + 135	Fludarabine and cyclophosphamide / cyclosporine (cyclosporine (Cyclosporine capsules) capsules), MMF Switched to sirolimus due to TMA	Mild acute skin GvHD	Parainfluenza virus	OI		Methyl prednisone 2mg/kg/day for 7 days followed by methyl prednisone 500 mg for 5 days	Death 7 months after BMT

ATG: Anti-thymocyte globulin; BMT: Bone marrow transplantation; CR: Complete response; DAT: Days after transplantation; F: Female; M: Male; MMF: mycophenolate mofetil (CellCept), NA: Not available; OI: Orotracheal intubation; TBI: Total bone marrow irradiation; TMA: thrombotic microangiopathy.

## Methods

A literature review was performed of cases of acute fibrinous organizing pneumonia in allo-BMT patients. The PubMed, Embase, Lilacs, and Cochrane databases were searched using the following terms “acute fibrinous and organizing pneumonia” AND “hematopoietic cell transplantation”. Table 2 presents reports of acute fibrinous and organizing pneumonia following allo-BMT between 2009 and 2024 [7, 8, 9].

## Case report

A 62-year-old female patient was diagnosed with acute myeloid leukemia with low cytogenetic risk (normal cytogenetics; *NPM1* positive, *FLT3ITD*/*TKD* negative). She achieved complete response after receiving induction chemotherapy using the 7 + 3 regimen (cytarabine (Cytarabine) 100 mg/m^2^ Days 1–7 and daunorubicin 90 mg/m^2^ Day 13) followed by three cycles of consolidation with high doses of cytarabine (Cytarabine). Six months after remission, molecular relapse (*NPM1*) was diagnosed. She underwent two cycles of azacitidine (75 mg/m^2^ Days 1–5) and venetoclax (400 mg/daily), again achieving complete response. She underwent allo-BMT with a reduced intensity conditioning regimen (fludarabine, cyclophosphamide, total body irradiation 4 Gy). The donor was her 33-year-old son, with 6 × 10^6^ CD34^+^ cells/kg from a peripheral blood source. As GvHD prophylaxis, she received cyclophosphamide, cyclosporine (cyclosporine (cyclosporine (Cyclosporine capsules) capsules) capsules) and mycophenolate mofetil (CellCept) post-transplant.

As post-infusion complications, she presented severe bloodstream sepsis due *to Rothia sp* and *Corynebacterium*, diarrhea due to enteropathogenic *Escherichia coli* and acute kidney injury requiring dialysis support for seven days. She presented late neutrophil engraftment on the 38th day after transplantation. The MMF was suspended on Day +30. She presented mild acute GvHD of the skin which was successfully treated with topical corticosteroid therapy.

**Figure 1:**
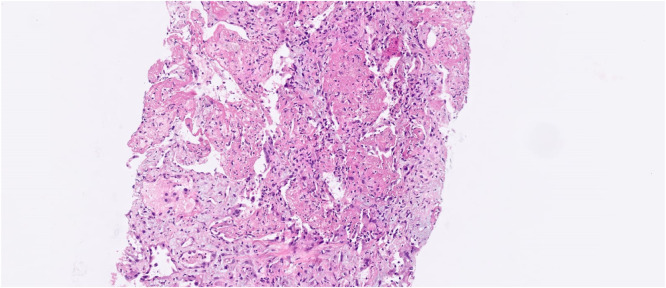
Fibrinous and organizing interstitial pneumonia with neutrophilic exudate - histological sections of the lung parenchyma showing moderate to severe exudate fibrinoleukocyte with frequent neutrophils and areas of neutrophilic debris. Presence of fibrinoid aggregates in organization in the alveolar spaces, with histiocytes containing hemosiderin. Pulmonary interstitium with mild edema and immature fibroplasia (focal myofibroblastic aggregates). No granulomas or malignancy were identified in the sample. Acid-resistant bacilli, bacteria and fungi by Ziehl-Neelsen stains, Brown-Breen, PAS and Grocott were negative. The immunohistochemical investigation of Herpes, CMV and Adenovirus viruses, resulted negative.

Four months after transplantation, she was hospitalized due to cough and dyspnea. A computed tomography (CT) scan showed diffuse ground glass opacities and alveolar consolidations compatible with organizing pneumonia. A bronchoalveolar lavage was performed without evidence of pathogenic agents by molecular and usual microbiological and culture techniques. Prednisone (1 mg/kg) was started, together with fluticasone, montelukast and azithromycin three times a week. The patient was discharged after two weeks and continued as outpatient for one month until she was readmitted due to worsening of the pulmonary symptoms. Another bronchoalveolar lavage was then performed that proved positive for parainfluenza virus in a respiratory virus polymerase chain reaction (PCR) panel (which had not been performed with the previous bronchoalveolar lavage). A CT scan showed worsening of the lung parenchymal opacities, and the patient had worsening of respiratory symptoms and hypoxemia. Given her clinical and radiological deterioration and the fact that the new bronchoalveolar lavage only revealed the presence of parainfluenza virus, a lung biopsy was deemed necessary for better etiological definition approximately one week after hospitalization. A CT-guided lung biopsy revealed moderate to severe fibrinoleukocyte exudate, frequent neutrophils, and areas of neutrophilic debris. The biopsy also showed organizing fibrinoid aggregates in the alveolar spaces, as well as histiocytes containing hemosiderin. These findings were compatible with fibrinous and organizing interstitial pneumonia (Figure 1).

The patient presented progressive hypoxemic respiratory failure, requiring mechanical ventilation ten days after admission. Methylprednisolone (2 mg/kg/day) was initiated for seven days followed by methylprednisolone pulse therapy (500 mg/daily) for five days with transient, non-sustained clinical improvement. A dose of Rituximab (375 mg/m^2^) was then administered in the context of clinical refractoriness to corticosteroids. Despite intensive support and medication, the patient was refractory to treatment and died of intractable hypoxemic respiratory failure, approximately one month and ten days after admission and seven months after the allo-BMT.

## Discussion

Table 1 shows the clinicopathological differences between the most well-known entity, OP, and AFOP. OP is characterized by intra-alveolar granulation tissue with myofibroblasts and connective tissue, while AFOP exhibits intra-alveolar fibrin aggregates with patchy distribution, organizing connective tissue, mild interstitial inflammation, and type 2 pneumocyte hyperplasia. The main clinical difference is that AFOP typically follows a much more aggressive and acute course. In the present report the diagnosis of AFOP was made in the pathology department by a pathologist specialized in respiratory diseases, who highlighted a pattern similar to that of organizing pneumonia (OP) but with some striking differences (mainly the presence of intra-alveolar fibrin deposits). The case was discussed in a multidisciplinary meeting involving a pulmonologist, radiologist and pathologist, as is recommended for the evaluation of interstitial lung diseases. This diagnosis is, indeed, challenging for pathologists without expertise in interstitial lung diseases.

To date, four cases of AFOP following allo-BMT have been described in the literature, with patient ages ranging from 25–62 years. Compared to older patients, those under the age of 60 had better outcomes, and patients diagnosed before Day +100 generally had a better response to treatment and more favorable clinical outcomes [8,9]. Regarding hematological diseases, two of the patients had leukemia and the other two had lymphoma. All underwent allo-BMT and subsequently developed AFOP, with corticosteroid therapy as part of the initial treatment in all four cases [8,9].

Simmons et al. described AFOP as a disease with a very aggressive course, associated with a high level of morbidity [9]. They reported the case of a 52-year-old male patient diagnosed with angioimmunoblastic T-cell lymphoma who developed AFOP on Day +325 after allo-BMT. He presented with a late form of AFOP, concomitant with chronic GvHD of the skin and eyes. The clinical presentation was severe, requiring mechanical ventilation and high-dose immunosuppression with methylprednisolone (250 mg q6h) and tacrolimus. Due to the refractoriness of the condition, etanercept (25 mg twice weekly for eight doses) was introduced. The authors suggested that the response to etanercept could highlight the role of tumor necrosis factor-alpha in a cytokine-modulated immune response causing inflammation and fibrosis [9].

Only one of the reports mentions concomitant GvHD (acute or chronic). In the current case, the patient presented with acute skin GvHD, which was resolved with topical corticosteroid therapy; AFOP developed after the resolution of the GvHD.

As AFOP after allo-BMT is a rare pulmonary disease manifestation, no consensus exists as to the optimal therapeutic approach. Beasley et al. reported a 40 % mortality rate for AFOP following allo-BMT, and severity markers such as the need for mechanical ventilation were present in all patients [3,7, 8, 9]. With improvements in antibiotic prophylaxis, infection-related mortality has decreased, making noninfectious pulmonary complications increasingly significant as causes of transplantation-related mortality. These complications are prevalent in 30–40 % of patients, with an overall mortality of 30 % [10, 11, 12]. Unfortunately, the underlying pathophysiology of many of these conditions remains poorly understood. While corticosteroids and immunosuppressive therapy remain the cornerstone of treatment for most noninfectious pulmonary complications after allo-BMT, the poor outcomes highlight the need for more effective and targeted therapies, as well as improved strategies for mitigation and prevention [13].

Concomitance of AFOP with infectious agents were not present in any of the reported cases. In the present case, the parainfluenza virus was isolated through the Respiratory Virus PCR Panel for the bronchoalveolar lavage. Although this viral infection alone would not account for the histopathological findings, we hypothesize that the presence of the virus may have triggered the development of AFOP.

Since AFOP is characterized by nonspecific radiological findings and specific histopathological features, a lung biopsy should be considered for patients with severe noninfectious respiratory failure after allo-BMT, especially when the cause is unclear or when a clinical diagnosis is elusive. Early initiation of corticosteroids is crucial for better outcomes. Other immunosuppressive agents may be considered, but given the scarcity of literature, further research is needed to optimize management strategies and improve outcomes.

## Conclusion

AFOP is a rare and severe complication following allo-BMT. Early diagnosis, histopathological confirmation, and prompt initiation of corticosteroid therapy are critical for improving outcomes. Patients diagnosed before Day +100 generally have a better response to treatment and more favorable clinical outcomes. The need for a more effective and targeted treatment strategy remains an unmet challenge in managing this condition.

## Conflicts of interest

None.
